# Recent Advances in the Development of Biosensors for Malaria Diagnosis

**DOI:** 10.3390/s20030799

**Published:** 2020-02-01

**Authors:** Francis D. Krampa, Yaw Aniweh, Prosper Kanyong, Gordon A. Awandare

**Affiliations:** 1 West African Centre for Cell Biology of Infectious Pathogens (WACCBIP), University of Ghana, P.O. Box LG 25, Legon, Accra, Ghana; aniweh@gmail.com (Y.A.); p.kanyong@waccbip.org (P.K.); gawandare@ug.edu.gh (G.A.A.); 2 Department of Biochemistry, Cell & Molecular Biology, University of Ghana, P.O. Box LG 54, Legon, Accra, Ghana; 3 Department of Chemistry, University of Oxford, South Parks Road, Oxford OX1 3QZ, UK

**Keywords:** malaria biomarkers, biosensors, clinical diagnosis, medical devices, biosensing

## Abstract

The impact of malaria on global health has continually prompted the need to develop more effective diagnostic strategies that could overcome deficiencies in accurate and early detection. In this review, we examine the various biosensor-based methods for malaria diagnostic biomarkers, namely; *Plasmodium falciparum* histidine-rich protein 2 (PfHRP-2), parasite lactate dehydrogenase (pLDH), aldolase, glutamate dehydrogenase (GDH), and the biocrystal hemozoin. The models that demonstrate a potential for field application have been discussed, looking at the fabrication and analytical performance characteristics, including (but not exclusively limited to): response time, sensitivity, detection limit, linear range, and storage stability, which are first summarized in a tabular form and then described in detail. The conclusion summarizes the state-of-the-art technologies applied in the field, the current challenges and the emerging prospects for malaria biosensors.

## 1. Introduction

Malaria remains an important parasitic human disease globally, which is transmitted via the bite of female *Anopheles* mosquitoes. The greatest burden of the disease is in the tropical and subtropical regions of the world [1,2]. The disease causes high economic burden to the countries that are endemic, mostly, developing countries. The etiologic agent is an Apicomplexan protozoan of the genus *Plasmodium*. Six species of this genus, namely, *Plasmodium falciparum, Plasmodium malariae, Plasmodium knowlesi, Plasmodium ovale* (*P. ovale curtisi* and *P.ovale wallikeri*), *Plasmodium cynomolgi* and *Plasmodium vivax* are known to cause infection in humans. As the World Health Organization (WHO) sets the goal for malaria elimination by 2030 [3], the aim can only be achieved when all cases are accurately diagnosed and treated appropriately. Some of the endemic communities still lack access to routine testing in suspected cases. For example, in 2018, only 74% of patients suspected to have malaria, excluding undocumented cases, had access to diagnostic tests in public health facilities [2]. A total of 228 million cases were recorded worldwide during this period out of which 405,000 mortalities occurred [4].

Different control strategies have been effective, but limited by ineffective early diagnostic tools for detection, especially, at low parasitemia and surveillance in low-transmission settings. The ability to detect asymptomatic individuals will greatly impact on transmission dynamics, malaria control, and possibly towards elimination. Diagnostic testing may help health service providers to further investigate other aetiologies of febrile illnesses; prevent severe disease and probable death; reduce the presumptive use of antimalarial drugs and associated side-effects; and mitigate against the rapid emergence and spread of drug resistance. It could also reduce the pool of individuals who can contribute to malaria transmission [5].

To date, many technologies have attempted to circumvent the challenges in malaria diagnostics with technologies that address point-of-care needs and early stage asymptomatic detection. In this review, we first comprehensively summarize the biomarkers targeted during the course of malaria with emphasis on the importance of sensitive early detection. Next, we provide an overview of the recent advances in biosensor technologies for the detection of the most targeted biomarkers, focusing on: development, analytical performances, and suitability for point of care testing. The prevailing challenges and future outlook of the use of these technologies in the field are also highlighted.

## 2. Parasite Development in Humans, Biomarkers, and Diagnosis

**Figure 1:**
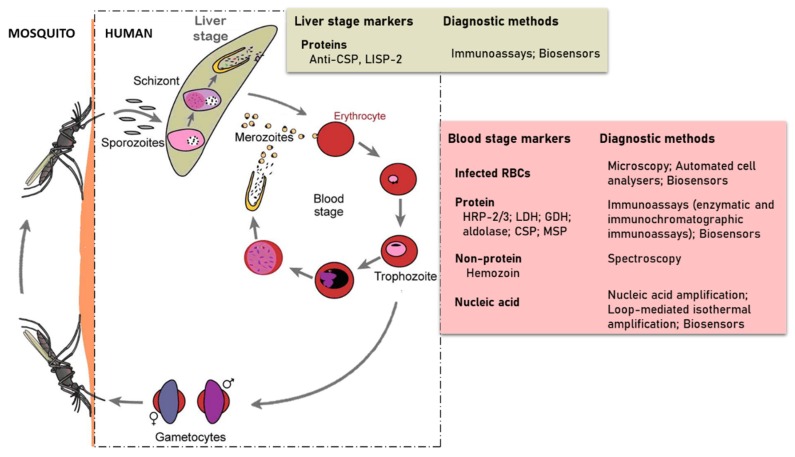
Developmental cycle of human *Plasmodium* species (redesigned from Scherf et al. 2008) in a mammalian host and the strategies used in detecting parasite specific markers.

The developmental cycle of *Plasmodium* species that infect humans is briefly illustrated in Figure 1 [6]. The cycle begins with the injection of sporozoites into the host’s circulation by an infected female *Anopheles* mosquito. The sporozoites then target and enter hepatocytes where they multiply and differentiate into merozoites. This stage of the parasite life cycle is known as pre-erythrocytic. In infections involving *P. vivax* and *P. ovale*, dormant forms of the liver stage, called hypnozoites may persist in the liver [7] and cause relapse of the infection, thereby making it difficult to eradicate. The pre-erythrocytic stage is essential in the establishment of malaria infection. This stage is asymptomatic, however, it is difficult for diagnostic tools to detect sporozoites because hepatocytes invasion occurs within 30–45 min after sporozoites are inoculated by the infected mosquito [8,9]. This short time and low numbers of sporozoites injected leaves little time for their detection. Some efforts in finding biomarkers for detection of early liver stage or the dormant form have identified the *Plasmodium* liver-specific protein 2 (LISP2) [10,11]. The sporozoite surface circumsporozoite proteins (CSP), which functions to interact with receptors on the hepatocyte, has also been targeted for diagnostic potential [12].

The erythrocytic stage of infection begins when merozoites released from the invade red blood cells (RBCs) and grow from the rings to trophozoites and schizonts stages of development. Schizonts egress to release merozoites that continue the cyclical asexual cycle. In the process, some of the parasites differentiate into gametocytes to begin the sexual phase of the life cycle. The gametocytes are taken up by female Anopheles mosquitoes during blood meal. This subsequently develops in the midgut through to ookinete and their transition into the salivary glands as sporozoites ready to be injected during blood meal to initiate infection in humans.

Various parasite detection methods have been employed over the years in diagnosing malaria cases. It is ideal to detect infection at the erythrocytic stage because of exponentially elevated parasite numbers, abundance of nucleic acid markers or the production of soluble antigenic proteins that can illicit immune responses. Using a microscope and efficient staining of peripheral blood, popularly Giemsa-stained thick and thin blood films, parasitized RBCs can be visualized and the different parasite species distinguished morphologically in: ring, trophozoite, schizont, and gametocyte. Although microscopy offers advantages such as good sensitivity and the capacity to determine parasitemia and the type of species, it is time-consuming and requires a highly skilled microscopist. Cases are sometimes misdiagnosed or undetected due to poor sensitivity at low parasitemia, which lead to inappropriate and/or delayed treatment [13,14]. These limitations associated with the routine use of microscopy have led to the development of alternative diagnostic methods such as polymerase-chain reaction (PCR)-based nucleic acid amplification tests that target specific genes or transcriptomes of the parasite at the erythrocytic stage. The commonly targeted genes or RNA transcripts and some recent advances in nucleic acid-based techniques have been extensively reviewed [15,16,17,18]. However, these alternative methods tend to be expensive, require trained personnel, lengthy turnaround time, and a level of sophistication that is not suitable for uptake in rural and poor healthcare settings [19].

The detection of a variety of other parasite-specific biomarkers including but not limited to histidine rich proteins 2 and 3 (HRP-2/3), lactate dehydrogenase (LDH), glutamate dehydrogenase (GDH), aldolase, merozoite surface protein 3 (MSP-3), and the biocrystal hemozoin have been explored order for a faster and easier diagnosis in the field. An extensive list of these biomarkers, as well as their metabolic role and clinical relevance, occurrence, genetic and structural organization, and kinetic parameters, have been discussed [1,20,21] and a brief overview is presented in subsequent sections of this review.

Immunochromatographic tests have become popular for point-of-care (POC) diagnosis of malaria [13,22]. Commonly called a ‘rapid diagnostic test’ (RDT), it is based on a lateral flow immunoassay technique integrated into a cassette for single-step, cost-effective, simple and fast detection of parasite specific biomarkers. These attributes have made the RDTs immensely popular in the field for POC application since its introduction, with the distribution reaching a global estimate 1.92 billion RDT units between 2010 and 2017 [23]. Africa is the biggest consumer with more than 80% of the total RDT sales in 2017 alone (223 million out of the 276 million units) [2].

*Plasmodium falciparum* histidine rich protein 2 (PfHRP-2) is the main target for RDTs that detect *P. falciparum* infection. A variant of LDH, the pan-specific LDH (pLDH) and pan-specific aldolase common to all species are used in combination with the PfHRP-2 either for *P. falciparum* alone or for mixed infection [24].

Although RDTs have dominated point-of-care-tests (POCT) for malaria, there have been major concerns about the stability and performance relating to sensitivity and specificity which constrains their impact [25,26,27]. Currently, the commercially available RDTs are about 1000-fold lower in sensitivity than alternative laboratory-based techniques [28,29,30], thus they do not provide the sensitivity and quantitation comparable to the gold-standard microscopy or PCR [29]. Studies have reported that RDTs which incorporate pan-aldolase have poor sensitivity due to low expression of the enzyme by parasites. As a result, only few RDTs combine pan-aldolase/PfHRP-2 [31,32]. Similarly, poor sensitivity in pLDH-based RDTs are often associated with low parasitemia and high tropical temperature [33]. This gap and limitations necessitate the need to develop other diagnostic technologies with improved sensitivities [34,35], while ensuring simplicity, robustness, and cost-effectiveness. Some of these recent advances have included chip-based microfluidics, surface plasmon resonance and biosensors, many of which have achieved comparable sensitivities with traditional diagnostic methods.

## 3. Biosensors for the Detection of Malaria Biomarkers in Clinical Samples

Biosensors and immunosensors have experienced unprecedented growth in recent years and seem to be the most promising sensing tools with several analytical benefits and cost efficiency [36,37]. This growth has been driven in part by the surge in demand for POC devices in clinical diagnosis where biological sensing is integrated with microelectronics to form portable analytical devices. To date, nearly sixty years after the first biosensor for glucose detection, the technology has been widespread in several fields of analyte detection [38]. Glucometers have evolved enormously, receiving vast commercial success [39] whereas biosensors for other diseases have been limited to experimental research. Among the types of sensors, electrochemical biosensors have received considerable interest in clinical diagnostics owing to key advantages in their design, assay simplicity, and superior analytical performance over conventional laboratory methods [40,41]. These qualities make them suitable for POC application amidst efforts to improve and miniaturize electrochemical systems for portable devices.

Most attempts to create miniaturized electrochemical devices for on-site analysis have applied screen-printed electrodes (SPE) as transducers and various nanomaterials as signal amplification strategies to improve the assay sensitivity [42,43,44,45]. Electrochemical immunosensors have been commonly applied to malaria diagnostic research given the benefits of low detection limits, wide linear response range, stability and reproducibility [46]. The strategies for detection comprise either a labelled assay in which apply amperometry and colorimetry or an impedimetric strategy, attractive for highly sensitive label-free detection [47,48]. Only a few potentiometric techniques have been reported [49]. The performances of selected biosensors reported for malaria biomarkers detection is summarized in Table 1. The choice of PfHRP-2 and LDH is still predominant, similar to RDTs. However, there is an increase in preference for pLDH possibly due to the persistence of PfHRP-2 antigenemia for several weeks after parasite clearance [50] and reports of mutant strains from Africa and Asia with deleted *PfHRP-2* genes [51,52,53].

### 3.1. Detection of PfHRP-2 in Clinical Samples

Histidine-rich protein 2 (HRP-2) is specific to *P. falciparum* (PfHRP-2) and is secreted into peripheral blood during parasite growth and development where it plays a role in heme detoxification. The antigen’s widespread application in electrochemical and optical immunosensors is due to copious expression levels throughout the parasite life cycle. Although primarily abundant in blood, trace amounts can be found in cerebrospinal fluid, urine, and saliva of infected patients [75,76], which offer an opportunity for non-invasive testing. Nonetheless, blood is preferred because of small sample volumes required to target the antigen. Painless testing has attractive public health benefits of voluntary testing and participation in screening programs geared towards malaria control [77]. However, only a few publications have attempted urine or saliva analysis as noninvasive malaria diagnostics [78].

Electrochemical techniques have been shown to outperform optical methods in many modelled POC tests. Nanoparticles, primarily gold (AuNP) have been adopted in signal amplification for amperometric immunosensors [79,80,81]. Their small size and ease of immobilizing bioconjugate probes allow for increased surface concentration of enzyme-tagged detection antibodies, hence higher signals from the catalytic reaction of enzyme and substrate. Sharma et al. were first to report an electrochemical immunosensor to detect PfHRP-2 in blood by amperometry [65]. The disposable immunosensor utilized multi-walled carbon nanotubes (MWCNTs) and gold nanoparticles (Nano-Au) to modify screen printed electrodes (SPE); resulting in Nano-Au/MWCNT/SPEs onto which rabbit-derived anti-PfHRP-2 were immobilized as capture antibodies. A sandwich enzyme-linked immunosorbent assay format was employed for the biosensor with alkaline phosphatase (ALP)-conjugated antibodies. Amperometric measurements were applied using ALP hydrolysis of 1-naphthyl phosphate. The Nano-Au/MWCNT/SPE had a limit of detection (LoD) of 8.0 ng/mL (compared to 80.0 ng/mL for bare SPE and 20.0 ng/mL for MWCNT/SPE) (Table 1). This enhanced performance was attributable to the synergistic effect of MWCNTs and AuNP. More importantly, the immunosensor had a superior analytical performance compared with a commercial immunochromatographic lateral flow test in the analysis of microscopy positive patient sample (sensitivity: 96% vs. 79%, specificity; 94% vs. 81% respectively).

In assessing exposure to malaria parasites, recombinant PfHRP-2 was used as a recognition element for anti-PfHRP-2 antibodies in an amperometric immunosensor for early stages of malaria and at low parasitemia [82]. SPEs were modified with alumina sol-gel (Al_2_O_3_) and AuNP to obtain AuNP/Al_2_O_3_ sol-gel/SPE after which PfHRP-2 was bound. Rabbit anti-PfHRP-2 and anti-rabbit IgG-ALP conjugate were directed against capture antigens and the analytical responses determined by amperometry. In comparison to ‘gold standard’ microscopy, the immunosensor exhibited a sensitivity of 92% and a specificity of 90%. In another study that detected monoclonal antibodies to recombinant PfHRP-2 (MoaPfHRP-2), a gold chip was pre-treated with 4-mercaptobenzoic to immobilize recombinant PfHRP-2, then monitored for interactions between the antigen and antibody [68]. Label free surface plasmon resonance (SPR) screening of the interaction between the recombinant protein and target antibody produced a LoD of 5.6 pg (Table 1).

Magnetic nanoparticles (MNPs) have also been applied in the development of a highly sensitive malaria immunosensor. Anti-HRP-2 was covalently attached to MNPs as capture elements and a second monoclonal antibody that binds a different epitope of the target antigen was labelled with horse radish peroxidase (HRP) to provide an electrochemical signal [67]. The anti-HRP-2-MNPs were captured onto a magnetic graphite-epoxy composite electrode incubated with HRP-2-spiked serum and anti-HRP-2-HRP in a sandwich assay format. Amperometric measurements produced an LoD of 0.36 ng/mL (Table 1), much lower than Sharma et al. [65] reported. Translating this strategy to the field would require magnetic supports for electrodes [67].

**Figure 2:**
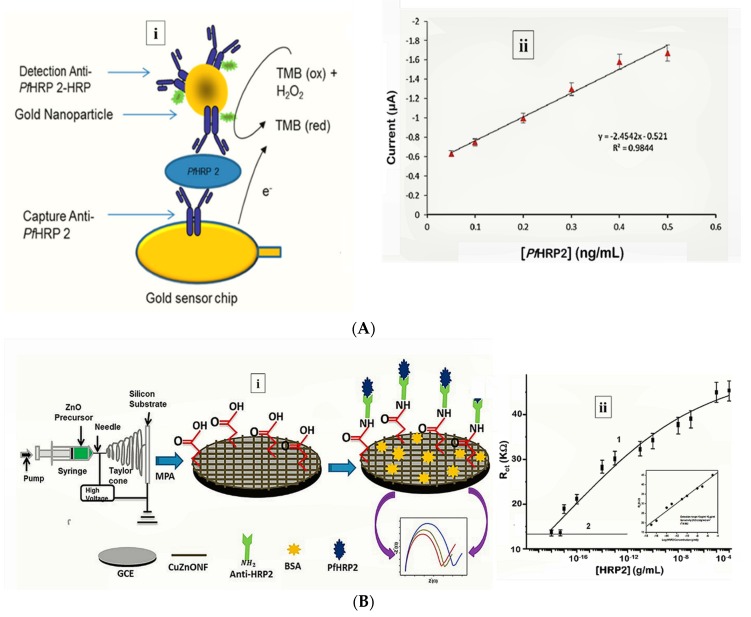
Strategy for (**A**) labelled amperometric [60] and (**B**) label free impedimetric [57] electrochemical detection of PfHRP-2. (**A**) (**i**) Gold nanoparticle amplified sandwich assay and (**ii**) plot of chronoamperometric response of PfHRP-2 detection in spiked serum (0.05–0.5 ng/mL PfHRP). Reprinted from Hemben et al. [60] with permission from MDPI. (**B**) (**i**) CZnONF dispersions drop-casted onto GCE followed by chemisoption of anti-HRP2 unto MPA treated electrodes and (**ii**) calibration curve of impedimetric responses obtained after incubating GCE/fCuZnONFs/Anti-HRP2 biosensor with varying concentrations of PfHRP2 (10 ag/mL–10 µg/mL). (Adapted from Paul et al. [57] with permission from Elsevier).

More recently, Hemben et al. used anti-PfHRP-2 monoclonal antibodies to capture PfHRP-2 at the surface of a screen printed gold electrode (Table 1) [60]. The captured antigen was targeted with HRP-labelled antibodies and the quantification of PfHRP-2 derived from the substrate (TMB-H_2_O_2_)-enzyme reaction by amperometry (Figure 2A). The LoDs in buffer and spiked human samples were determined as 2.14 ng/mL and 2.95 ng/mL, respectively. Labelled antibodies were subsequently conjugated to gold nanoparticles (AuNP) to amplify the sensor signal which improved the sensitivity and LoD in buffer (36.0 pg/mL) and spiked serum samples (40.0 pg/mL).

While biosensing platforms for most disease biomarkers tend to rely on antibodies as capture molecules, a challenge with immunoassays is related to antibody stability, a prerequisite. Some attempts to circumvent these drawbacks have included genetic manipulations that improve the stability and shelf-life of antibodies and the use of synthetic alternatives such as aptamers. For example, researchers cloned and expressed cDNA fragments encoding the variable domains (VL-CL and VH-CH1) of two monoclonal antibodies against PfHRP-2 (F1546 and F1110) in *Escherichia coli* [83]. The recombinant Fab fragments showed similar binding properties to those of the parental monoclonal antibodies (mAb). This approach proposes a cost-efficient alternative to large-scale antibody production for diagnostic application with the opportunity of engineered antibody fragments with improved affinity, stability, and resistance to denaturation even with prolonged storage in uncontrolled temperatures in the field.

Besides using molecular labels and nanoparticles for improved diagnostics, some assays can probe the intimate recognition between the receptor and target alone. The benefits of using such label-free formats include a reduction in the assay complexity, preparation time, and analysis cost as it eliminates potentially confounding chemical labels. These strategies are better suited to field applications and under resourced settings where laboratories and skilled personnel are unavailable. A label-free a piezoelectric immunosensor for PfHRP-2 was designed by. applying mixed self-assembled monolayers (SAMs) of thioctic acid and 1-dodecanethiol on gold quartz crystal microbalance (QCM) Anti-PfHRP-2 antibodies were covalently immobilized unto the SAM-modified electrodes via EDC-NHS activation and the frequency change resulting from binding of different concentrations of PfHRP-2 measured [84]. The immunosensor exhibited a LoD of of 12.0 ng/mL with a linear range of 15.0–60.0 ng/mL for analysis of PfHRP-2 in buffer. This was higher than their previously reported amperometric immunosensor (8 ng/mL [65] vs. 12ng/mL [84]), and weaker responses were observed for PfHRP-2 concentrations lower than 25.0 ng/mL. However, application of the sensor to clinical samples produced comparable sensitivities with a commercial immunochromatographic test (ICT) kit (NOW^®^ Malaria, Binax, Inc., Scarborough, Maine, USA).

An indicator displacement assay (IDA) was used to detect and quantify HRP-2 in sera [62]. The label-free spectrophotometric method did not require any biorecognition elements and was based on the color change of murexide either in complex with nickel (Ni^2+^) or free in solution. In the IDA, competition between HRP-2 and Ni^2+^ displaces murexide from the murexide-Ni^2+^ complex. The resultant color intensity was proportional to free unbound murexide is measured to quantify HRP-2. The assay had LoD of 30.0 ± 9.6 nM (dynamic range of 10–100.0 nM) without any interfering signals from serum proteins (Table 1).

Electrochemical impedance spectroscopy (EIS)-based methods present with numerous advantages that make them suitable candidates for POC application [85]. Their high sensitivity is evident from lower LoDs they tend to produce compared with other electrochemical methods. The lowest LoD yet reported for malaria was achieved by impedimetric detection of PfHRP-2 [57] (Table 1). In the sensor design, copper doped zinc oxide nanofibers (CZnONF) was drop-casted on glassy carbon electrode (GCE/CZnONF) followed by SAM modification and chemisorption of anti-PfHRP-2 (Figure 2B). The highly sensitive nanosensor (28.5 kΩ/(g/mL)/cm^3^) attained a detection limit of 6.8 ag/mL. The authors subsequently reported a flexible chemiresistive immunosensor in which the transducer comprised a 1-dimensional MWCNT-zinc oxide (MWCNTs-ZnO) nanofiber drop-casted on micro gold electrodes [44] (Table 1). Capture antibodies, anti-PfHRP-2 were immobilized on MWCNTs-ZnO by EDC-NHS crosslinking and resistance changes (ΔR) measured to monitor the formation of PfHRP-2-anti-PfHRP-2 complexes. The device demonstrated good analytical performance as well as potential towards the development of POCTs with a linear response ranging from 10 fg/mL to10 ng/mL, LoD of 0.97 fg/mL and high specificity for PfHRP-2 over non-specific antigens.

### 3.2. Detection of pLDH in Clinical Samples

*Plasmodium* lactate dehydrogenase (pLDH) plays a catalytic role in the glycolytic pathway during the intraerythrocytic stages of *Plasmodium*. It is produced by metabolically active parasites within infected red blood cells (RBCs) [86,87,88] and has conserved catalytic residues in all *Plasmodium* spp. except in *P. knowlesi.* Unlike HRP-2, pLDH is indicative of a recent infection and is generally cleared within 24 h of parasite clearance; hence, it more reliable in identifying recent unresolved infections.

There seems to be a growing trend of aptamer-based sensors targeting pLDH [54,55,89]. Compared to antibodies, aptamers are smaller in size, thermostable, extended shelf life without functional degradation, affordable, easily synthesized and can be readily modified. It could, to an extent be an alternative remedy in overcoming difficulties associated with using antibody-based tests. The unit costs for malaria RDTs in many African countries falls within the unsubsidized range of USD 2.54–2.83. Yet, a study in Uganda revealed that consumers were willing to pay an average of USD 0.53 [90]. The prospects of some biosensors/aptasensors being estimated at 1 USD or less per test, proposes a significant reduction to the general healthcare costs in impoverished tropical regions where malaria is prevalent.

In a prospective tool for asymptomatic and early diagnosis of malaria, single-stranded DNA aptamers (pL1 aptamers) were used to target recombinant *Plasmodium falciparum* LDH (PfLDH) and *Plasmodium vivax* LDH (PvLDH) in buffer and in real samples [55] (Table 1). Impedance measurements for the interaction between pL1 and the target proteins demonstrated high sensitivity and specificity with LoDs measuring 108.5 fM (for PvLDH) and 120.1 fM (PfLDH). Native pLDH in clinical samples was also detected up to 1 parasite/µL.

**Figure 3:**
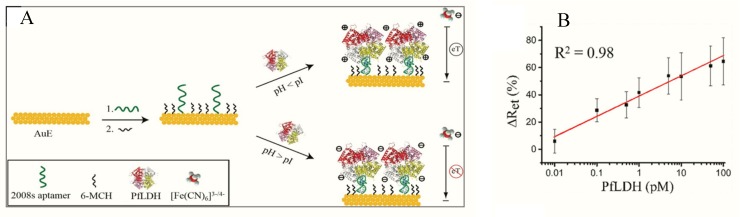
(A) Schematic representation of the PfLDH aptasensor and (B) calibration plot for 0.01 pM–10 nM PfLDH in 5 mM [Fe(CN)6]^3−/4−^ solution at pH 7.5. (Reprinted from Figueroa-Miranda et al. [89] with permission from Elsevier).

Figueroa-Miranda et al. immobilized 2008s aptamers on a SAM-modified gold electrode to bind pLDH down to the detection limit of 0.84 pM in buffer and 1.30 pM in blood (Figure 3, Table 1) [89]. The aptasensor had a detection range between 1 pM–10 nM and remained highly selective for PfLDH even in the presence of high concentrations of serum proteins and analogues of LDH from muse muscle. The thiol-gold covalent bonding from SAMs conferred high immobilization stability to the aptamer and could be regenerated and re-used for up to three times without loss of analytical performance. A major observation was the influence of isoelectric point (pI) of PfLDH on impedimetric responses which tended to increase where pH > pI and a decrease at pH < pI. A likely reason for this occurrence is attributable to a repulsion and attraction of the redox probe to the electrode surface.

**Figure 4:**
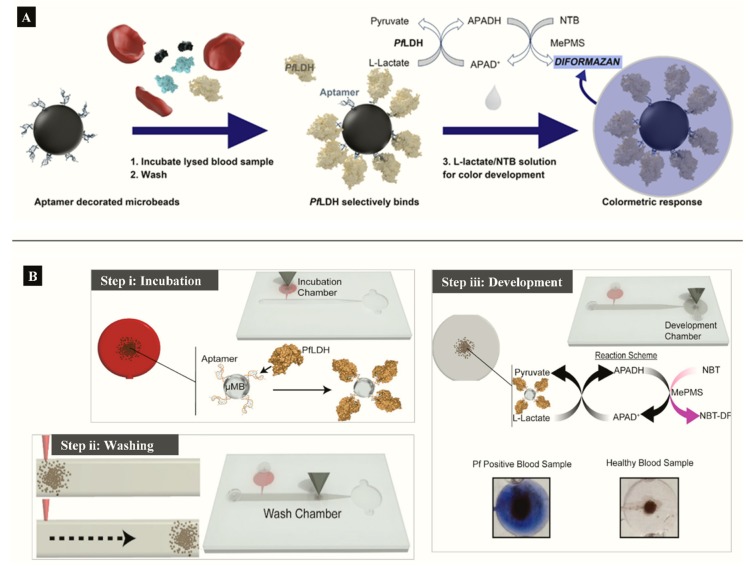
(**A**) APTEC biosensor based on capturing PfLDH and using its enzymatic activity to produce a colorimetric assay (Reprinted from Dirkzwager et al. [63] with permission from the American Chemical Society). (**B**) Working principle ((**i**) incubation, (**ii**) washing (**ii**) and (**iii**) color development) of a simple portable 3D-printed microfluidic device for diagnosis of malaria in clinical samples constructed from the APTEC assay (Reproduced from Fraser et al. [64] with permission from Elsevier).

Aptamer functionalized microbeads were used to determine the capture and measure the intrinsic enzymatic activity of LDH in a colorimetric assay (Figure 4A) [63]. In the aptamer-tethered enzyme capture (APTEC) assay, the beads conferred a wide surface area for analyte binding to produce a LoD of 4.9 ng/mL for recombinant PfLDH (Table 1). Further work integrated the APTEC assay into a portable microfluidic biosensor (Figure 4B) [64]. The platform resolved some of the assay’s initial limitations of large sample and reagent volumes while detecting *P. falciparum* with high specificity and sensitivity in cultures and clinical samples.

Fluorescently-labelled aptamers were adsorbed to molybdium disulphide (MoS_2_) nanosheets to develop a FRET aptasensor which selectively detected pLDH in a heterogeneous protein mixture [66]. The mechanism of the assay was based on a “capture-release” model whereby fluorescence of the aptamer is quenched upon aptamer-MoS_2_ nanosheets binding and restored in the presence of pLDH when the aptamer is released from the nanosheets. The attachment and detachment processes are facilitated by the high affinity between aptamers and pLDH. The sensor achieved LoD of 550.0 pM (Table 1).

Hemben et al. functionalized screen-printed gold electrodes (SPGE) with anti-pLDH antibodies and applied a sandwich assay format to detect pLDH [61]. The sensor initially achieved LoDs of 1.80 ng/mL in buffer and 0.70 ng/mL in serum. Application of colloidal AuNPs functionalized with HRP-labelled detection antibodies enhanced amperometric signals to LoDs down to 19 pg/mL (in buffer) and 23 pg/mL (in serum) (Table 1).

### 3.3. Detection of GDH in Clinical Samples

Ubiquitous enzymes such as glutamate dehydrogenases (GDH) in *Plasmodium* parasites play a role in glutamate catabolism and ammonium assimilation [1,91,92,93]. The enzyme is present throughout the sexual and asexual stages of the parasite development in significantly soluble quantities [94]. Several structural and kinetic distinctions between host and parasite GDH makes it potentially useful in targeting live parasites [95].

**Figure 5:**
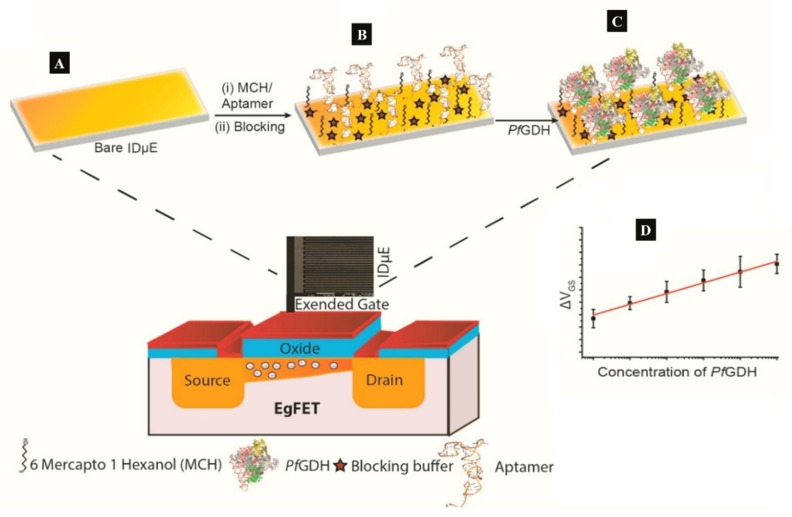
(**A**–**C**) Strategy for the label free aptaFET and **(D)** calibration curves obtained for PfGDH detection in serum. (Adapted from Singh et al. [49] with permission from Elsevier).

A label-free capacitive aptasensor was constructed by graftting thiolated ssDNA aptamer (NG3) specific to *P. falciparum* (PfGDH) on a gold electrode [59]. The sensor produced a LoD of 0.77 pM in serum with dynamic range 100 fM−100 nM (Table 1). Subsequently, the authors integrated the process into an extended gate field effect transistor (EgFET).The NG3 aptamers were immobilized on an inter-digitated gold microelectrodes (IDµE) and connected to the FET to construct a sensitive and stable miniaturized aptaFET biosensor (Figure 5) [49]. A benefit of FET-type systems is that, it enables for sensitive and simple electrochemical measurements without requiring a typical redox marker [96]. Following calibration curves in varying concentrations of PfGDH-spiked buffer and serum, a linear detection range of 100 fM–10 nM was obtained with LoDs in buffer and serum being 16.7 pM and 48.6 pM, respectively (Table 1). The FET-based potentiometric sensor was highly selective in the presence of analogous human and plasmodial proteins, making it suitable for analysis of real sample for malaria diagnosis.

### 3.4. Detection of Aldolase

Aldolase plays a key role in the glycolytic pathway of *Plasmodium* species where it catalyzes cleavage of fructose-1,6-bisphosphate into glyceraldehyde-3-phosphate and dihydroxyacetone phosphate [97]. Targeting aldolase as an antigens in malaria has been largely confined to ICTs, however, an evaluation of four aldolase- and LDH-based commercial ICTs found variations in specificity to *P. vivax* [98]. A probable reason why aldolase biosensors have not received much interest could be due to the poor sensitivity reported in aldolase ICTs. The genes encoding aldolase in *P. falciparum* and *P. vivax* are highly conserved [99], making it a poor marker of differential diagnosis. This adds on to the growing recommendations of paralleled detection of malaria antigens in test devices in order to maximize sensitivity and specificity while reducing the risk of misdiagnosis.

### 3.5. Detection of Hemozoin in Clinical Samples

At the erythrocytic stage of its life cycle, the malaria parasites digest about 60–80% of erythrocytic hemoglobin resulting in the formation of heme and polymerized to insoluble hemozoin crystallites [100]. Hemozoin is localized in parasite digestive vacuoles, therefore its presence in blood indicates a good marker of metabolically active *Plasmodium* parasites. The potential of surface-enhanced Raman spectroscopy (SERS) has been explored and shown to enhance the Raman signal of hemozoin by several folds [101]. Exposure of parasitized RBCs to a gold-coated butterfly wing as SERS substrate produced Raman shift within malarial hemozoin pigment whereas uninfected lysates did not. The spectral markers of hemozoin from infected RBC were detectable at the early-ring stage parasitemia levels of between 0.0005% and 0.005%. While enhancements of Raman signals occurs when hemozoin crystals are in direct contact with metal surfaces [102], another SERS method that applied synthesized silver nanoparticles inside parasites to achieve a close contact with hemozoin demonstrated an ultrasensitive hemozoin detection at 0.00005% parasitemia level in the ring stage (2.5 parasites/µL). These SERS methods have shown potential in early malaria diagnosis at low parasitemia levels, however, Raman spectrometers, and particularly those with high spectral resolutions, are expensive.

The paramagnetic properties of hemozoin crystals have been exploited for label-free detection using magnetic resonance relaxometry (MRR). In combination with a microfluidic setup, the MRR system achieved an accurate early detection at a parasitemia level of 0.0005% (Table 1) [74].

### 3.6. Detection of Other Relevant Malaria Biomarkers

Until date, most malaria biosensors have been principally confined to the established markers. Some other likely candidate targets have been speculated; heat-shock protein 70 (Hsp70), dihydrofolate reductase (DHFR)–thymidylate synthase (TS), heme-detoxification protein (HDP), glutamate-rich protein, hypoxanthine phosphoribosyl transferase, and phosphoglycerate mutase [103,104,105].

**Figure 6:**
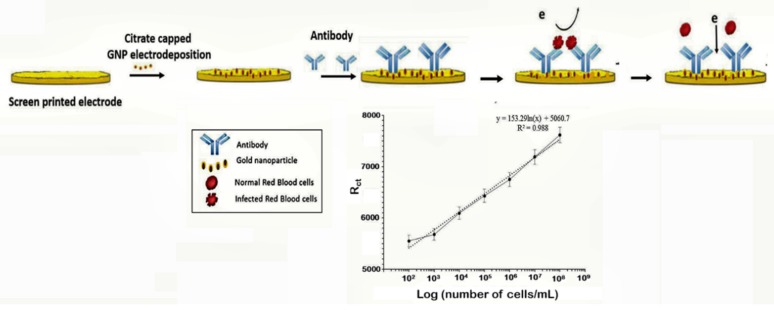
Schematic design of the impedimetric sensor malaria-infected RBCs and corresponding calibration plot. (Reprinted from Kumar et al. [47] with permission from the Royal Society of Chemistry).

The capture of parasitized RBCs has been proposed as an alternative in overcoming the paucity of known malaria biomarkers. Even at low parasitemia, the populations of parasitized RBCs are elevated, reaching about 10,000 cells/µL in 0.2% parasitemia and 250,000–500,000 infected cell/mL in 5–10% parasitemia [106]. Based on this knowledge, a novel microfluidic SELEX (I-SELEX) was implemented to identify a variant set of aptamers that distinctly bound different epitopes present on parasitized RBC surfaces [107]. In another study, researchers immobilized monoclonal antibodies on a AuNP modified screen-printed electrode as capture elements for malaria-infected cells [47] (Figure 6). Impedimetric changes caused by the interaction of monoclonal antibodies and parasitized RBCs distinguished infected from normal uninfected RBCs, measurable over a linear response concentration range of 10^2^–10^8^ cells/mL of infected RBCs (Table 1).

In another study, a highly sensitive and inexpensive biosensor was modelled to detect antibodies specific to *P. vivax* in serum (Table 1) [108]. Screen-printed carbon electrodes were coated with carbon nanotubes (CNTs) and EDC-NHS used to immobilize circumsporozoite protein (CSP) and thrombospondin related anonymous protein (TRAP). The assay detected anti-CSP and anti-*P. vivax* TRAP (anti-PvTRAP) antibodies directed against both antigens as low as 6–50 pg/L (concentrations in the range of 10–15 M) by means of EIS. Overall, the method demonstrated a promising approach to real-time probing of antibodies in serum directly without the need for sample preparation [108].

Nucleic acid markers have been exploited as reliable alternatives to proteins and antibodies in malaria diagnostics [109]. A DNA biosensor based on quartz crystal microbalance (QCM) was designed to targeted merozoite surface protein 2 (msp2) genes in *P. falciparum* [69]. The post-PCR label-free biosensor used avidin-biotin interactions to immobilize biotinylated probes complementary to the msp 2 gene unto gold QCM. Initial validation on laboratory strains showed probe-target binding at concentrations from 25–250 ng/mL and LoD of 0.025 ng/mL as well as genotyping potential (Table 1). Application of clinical samples confirmed no cross-reaction between species. In other studies, silver fabricated QCM platforms were applied to detect specific DNA fragments [110] and distinguish 18s rRNA gene of *P. falciparum* and *P. vivax* [72]. Both of these sensors were sensitive and could significantly differentiate species and mixed infections at the molecular level. Considering the hazards of UV visualizations and staining associated with agarose gel electrophoresis, the malaria gold/silver QCM promises a safer alternative. However, QCM methods still require prior amplification of genes which may not be feasible for POCT or immediate field uptake in under-resourced endemic areas where the capacity to perform PCR is lacking. A SERS-based method proposed by Ngo et al. to detect *P. falciparum* DNA fragments is suitable for automation and integration into portable molecular POCTs [71]. The assay comprised two complementary probes; a capture and reporter hybridized to magnetic beads and nanoratles (SERS tag) respectively, used to detect specific DNA in a sandwiched hybridization. The magnetic bead-DNA sequence-nanorattle formed in the presence of *P. falciparum* DNA target sequences is then concentrated for SERS measurement. The assay produced a LoD of 100 attomoles DNA (Table 1).

### 3.7. Multi-Panel Biomarker Arrays for Malaria Detection

Simultaneous analysis of biomarkers maximizes the use of samples and gives a wide range of results that significantly improves test accuracy. Increased throughput, reduced reagents/assay setup and less labor are among features that could decrease assay errors and make parallel testing ideal for healthcare delivery. Moreover, the benefit directly translates to convenient sampling and low cost to providers and patients at the same time ensuring optimum outcome. A combination of antigens and genes have been reported in ICT and nucleic-acid-based methods respectively and are discussed elsewhere [111,112,113]. Multiplexed malaria testing is aimed primarily at differential diagnosis between parasite species. The most common methods combine PfHRP-2/LDH or PfHRP-2/aldolase to distinguish passive/resolved or active infections as well as differentiate falciparum from non-falciparum malaria [114]. Other multiplexed systems involving malaria antigens aim to differentiate malaria from other febrile illnesses (malaria/typhoid [115] and malaria/dengue [116]) since febrile presentations tend to mimic malaria clinical course leading to over-diagnosis and presumptive treatment of malaria.

## 4. Conclusions and Future Perspective

The drawbacks of diagnostics in spite of the vast array of tests require more effective alternatives that is crucial in the long-term fight against/and probable elimination of malaria. Taking the tremendous success of glucometers into account, biosensors are a potential driving force in the emerging medical demands for onsite diagnostics in resource-limited settings. The growing interests in biosensor research and the progress made with the prototypes from published data implies that the technology could have a significant impact on malaria diagnosis regardless of being in nascent stages. These concepts, so far have offered ideal and desirable characteristics like high performance, low fabrication costs and easy operation with the aim of practicality in clinical diagnostics. The sensors discussed in this review present with impressively low detection limits that are superior to immune chromatographic dipstick which is usually between 2–16 nM. Overall, it can be argued that the slight increments in LoDs observed in serum samples relative to buffer are attributable to the complexity of serum that can hinder interactions between biorecognition molecules and target biomarkers. Given that a major challenge to malaria diagnosis is the lack of highly sensitive tools POCTs at low parasitemia, the impressive analytical performances that biosensors have demonstrated would allow for ultra-sensitive diagnosis of asymptotic cases and monitoring treatment with antimalarial drugs. To put this in perspective, randomized control trials that apply these technologies to a large number of clinical samples are needed in order to evaluate the practical usefulness. For easy translation of malaria biosensors to the field, aspects related to complexity and instrumentation which minimizes user intervention while ensuring low-cost are key areas which remains unclear. Studies are required to model self-contained devices as most biosensors presented rely on traditional immunoassay principles and involve multiple assay steps. Innovative technologies should take advantage of 3-D printing techniques and microfluidics to integrate prototype sensors into self-contained lab-on-chip models in readiness for field deployment.

The increase in preference for probing LDH in blood seems an amelioration in malaria diagnostics since its concentration correlates with parasite density and implies metabolically active parasites. This is in contrast to HRP-2 that either has delayed clearance or is absent in certain mutant strains. Furthermore, considering the advantages of electrochemical systems in POCTs, efforts towards improved system complexity could propose appropriate electrochemical systems for enzyme biomarkers like LDH, GDH, and aldolase that could be easily integrated into enzyme-based biosensors. For instance, substrates that are specific to these enzymes could be incorporated for direct enzymatic reaction detectable through electrochemical methods. Undoubtedly, the future of biosensors is being changed by the growing demands for novel biotechnologies in POC diagnostics. These innovations coupled with the capacity of multiplexing are necessary in fulfilling a major requirement of detection and species differentiation in the clinical diagnosis of malaria.

## Figures and Tables

**Table 1:** Summary of selected biosensors reporting detection of various malaria biomarkers.

Analytes	Sensing Technique/Response	Transducer	Biomarker	Receptor Molecule	LoD	Range	Response Time	Storage Stability	References
Antigens	Colorimetric	-	pLDH (PvLDH, PfLDH)	pL1 aptamer	8.3–8.7 pM (PvLDH) 10.3–12.5 pM (PfLDH)	NA	NA	NA	[54]
	EIS	Gold electrode	pLDH	pL1 aptamer	** 108.5 fM for PvLDH ** 120.1 fM for PfLDH	NA	NA	NA	[55]
	EIS	GCE	pLDH	P38 aptamer (90 mer ssDNA)	0.5 fM	NA	-	NA	[56]
	EIS	GCE	HRP-2	Anti-HRP-2 antibody	** 6.8 ag/mL.	10 ag/mL–10 mg/mL	NA	2 months (86.5%)	[57]
	Chemiresistive (electrical conductance)	-	PfHRP-2	Anti-HRP-2 antibody	0.97 fg/mL	10 fg/mL–10 ng/mL	NA	15 days (94.2%)	[44]
	-	-	PfHRP-2	Anti-PfHRP-2	0.025 ng/mL	0.01–10 ng/mL	-	-	[58]
	EIS	Gold disc electrodes	*Pf*GDH	ssDNA aptamer (NG3)	* 0.77 pM	100 fM–100 nM	NA	NA	[59]
	Potentiometric (FET)	Gold micro-electrodes	*Pf*GDH	ssDNA aptamer (NG3)	** 16.7 pM * 48.6 pM	100 fM–10 nM	5 s		[49]
	Amperometric	Gold-SPE	PfHRP-2	Anti-PfHRP 2 mAb	** 36 pg/mL * 40 pg/mL	NA	NA	NA	[60]
	Amperometric	Gold-SPE	pLDH	pLDH capture antibody	** 19 pg/mL * 23 pg/mL	-	-	-	[61]
	Spectrophotometric Indicator displacement medium	-	PfHRP-2	NA	30 ± 9.6 nM	10–100 nM	5 min	NA	[62]
	Colorimetric	-	PfLDH	2008s-biotin DNA aptamer	** 4.9 ng/mL	NA	<1h	2 months	[63]
	Colorimetric	-	PfLDH	2008s aptamer	-	NA	20 min	-	[64]
	Amperometric	SPE	PfHRP-2	Mouse anti-PfHRP-2 antibody	** 8 ng/mL	NA	NA	NA	[65]
	FRET	-	pLDH	Fluorescently-labeled aptamer (36 mer ssDNA)	** 550 pM	NA	NA	NA	[66]
	Amperometric magneto Immunosensor	-	PfHRP2	Anti-HRP2 IgM Antibody	0.36 ng/mL	0.35–7.8 ng/mL	NA	NA	[67]
Antibodies	SPR	Gold disc	Antibodies of *Pf.*	PfHRP2	** 5.6 pg for mAb	-	NA	NA	[68]
Nucleic acids	Quartz Crystal Microbalance	-	*Pf* msp2 gene	Biotinylated probe	≥0.025 ng/mL of target DNA	NA	NA	180 days	[69]
	Droplet Microfluidic Platform	-	*Pf* topoisomerase I	ds DNA substrate	NA	NA	NA	NA	[70]
	SERS Nanoplatform	-	Pf DNA sequences	Magnetic bead and nanorattle	100 attomoles	10^−11^–10^−10^ M	NA	NA	[71]
	Quartz Crystal Microbalance	Silver electrode	18s rRNA gene (Pf and Pv)	immobilized probe	-	-			[72]
Infected red blood cells	EIS	SPE	*Pf* infected RBCs	monoclonal antibody	-	10^2^–10^7^ cells/mL	NA	NA	[73]
	microfluidic separation and MRR	-	Infected RBCs	-	0.0005% parasitemia	-	-	-	[74]

LoD: limit of detection; * LoD: LoD in real samples; ** LoD: LoD in buffer; EIS: electrochemical impedance spectroscopy; FET: field effect transistor; FRET: fluorescence resonance energy transfer; GCE: glassy carbon electrode; MRR: magnetic resonance relaxometry; SPE: screen-printed electrode; SERS: surface-enhanced Raman spectroscopy; SPR: surface plasmon resonance; SWV: square wave voltammetry.
